# *rac*-3-{4-[(4-Nitro­benzyl­idene)amino]-3-phenyl-5-sulfanyl­idene-4,5-dihydro-1*H*-1,2,4-triazol-1-yl}-1,3-diphenyl­propan-1-one

**DOI:** 10.1107/S1600536811035197

**Published:** 2011-09-30

**Authors:** Wei Wang, Yan Gao, Chao Xu, Wen-peng Wu, Qing-lei Liu

**Affiliations:** a School of Perfume and Aroma Technology, Shanghai Institute of Technology, Shanghai 200235, People’s Republic of China; b School of Chemical Engineering, University of Science and Technology LiaoNing, Anshan 114051, People’s Republic of China

## Abstract

In the title mol­ecule, C_30_H_23_N_5_O_3_S, the 1,2,4-triazole ring is approximately planar (r.m.s. deviation = 0.006 Å), and forms dihedral angles of 66.0 (2), 65.1 (2), 30.1 (2) and 28.1 (2)° with the four phenyl rings. The phenyl ring of the benzyl group directly attached to the triazole ring is almost perpendicular to the nitro­phenyl ring, making a dihedral angle of 84.9 (2)°.

## Related literature

For the crystal structures of related 1,2,4-triazole-5(4*H*)-thione derivatives, see: Al-Tamimi *et al.* (2010 ▶); Fun *et al.* (2009 ▶); Gao *et al.* (2011 ▶); Tan *et al.* (2010 ▶); Wang *et al.* (2011 ▶); Zhao *et al.* (2010 ▶).
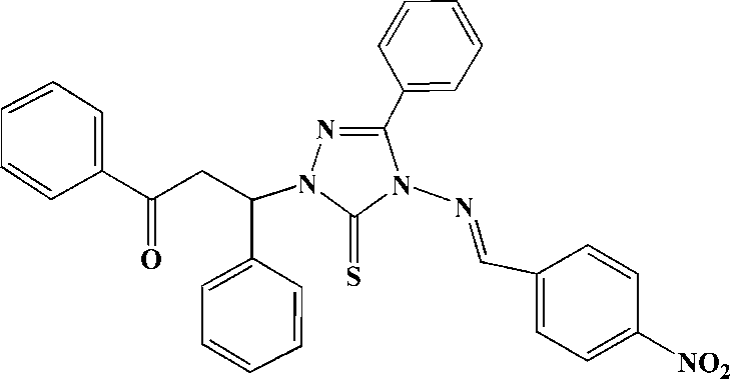


## Experimental

### 

#### Crystal data

C_30_H_23_N_5_O_3_S
*M* *_r_* = 533.59
Monoclinic, 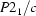

*a* = 13.3303 (13) Å
*b* = 21.832 (2) Å
*c* = 9.2773 (9) Å
β = 98.213 (3)°
*V* = 2672.2 (4) Å^3^
*Z* = 4
Mo *K*α radiation
μ = 0.16 mm^−1^
*T* = 113 K
0.20 × 0.18 × 0.12 mm

#### Data collection

Rigaku Saturn CCD area-detector diffractometer
Absorption correction: multi-scan (*CrystalClear*; Rigaku/MSC, 2005 ▶) *T*_min_ = 0.968, *T*_max_ = 0.981
27241 measured reflections
6372 independent reflections
5124 reflections with *I* > 2σ(*I*)
*R*_int_ = 0.040

#### Refinement

*R*[*F* ^2^ > 2σ(*F* ^2^)] = 0.049
*wR*(*F* ^2^) = 0.131
*S* = 1.10
6372 reflections
352 parameters
H-atom parameters constrained
Δρ_max_ = 0.33 e Å^−3^
Δρ_min_ = −0.25 e Å^−3^

### 

Data collection: *CrystalClear* (Rigaku/MSC, 2005 ▶); cell refinement: *CrystalClear*; data reduction: *CrystalClear*; program(s) used to solve structure: *SHELXS97* (Sheldrick, 2008 ▶); program(s) used to refine structure: *SHELXL97* (Sheldrick, 2008 ▶); molecular graphics: *SHELXTL* (Sheldrick, 2008 ▶); software used to prepare material for publication: *SHELXTL*.

## Supplementary Material

Crystal structure: contains datablock(s) global, I. DOI: 10.1107/S1600536811035197/zs2140sup1.cif

Structure factors: contains datablock(s) I. DOI: 10.1107/S1600536811035197/zs2140Isup2.hkl

Supplementary material file. DOI: 10.1107/S1600536811035197/zs2140Isup3.cml

Additional supplementary materials:  crystallographic information; 3D view; checkCIF report

## Figures and Tables

**Fig. 1.**
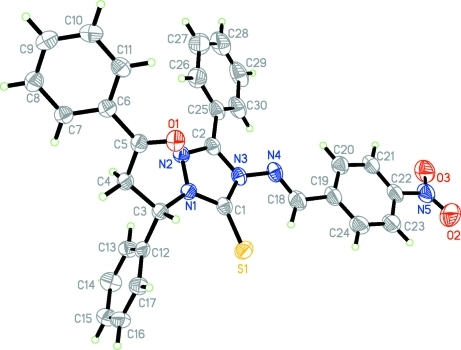
A view of a molecule of the title compound showing the atom-labelling scheme. Displacement ellipsoids are drawn at the 60% probability level.

## supplementary crystallographic information

### Comment

In a continuation of the structural study by our group of Mannich base derivatives synthesized by the reaction of amino heterocycles and aromatic aldehydes (Wang *et al.*, 2011), we present here the crystal structure of the title compound, C_30_H_23_N_5_O_3_S.

In this compound the bond lengths and angles are comparable with those reported in the related 1,2,4-triazole-5(4*H*)-thione derivatives (Al-Tamimi *et al.*, 2010; Fun *et al.*, 2009; Tan *et al.*, 2010; Wang *et al.*, 2011). The 1,2,4-triazole ring is planar with an r.m.s 0.0055 (2) Å and a maximium deviation of 0.0083 (2) Å  for atom N3. The C1 and C2 atoms of the 1,2,4-triazole ring show distorted C*_sp_*^2^ hybridization states with bond angles of 102.69 (13)° (N1—C1—N3); 131.16 (12)° (N3—C1—S1); 110.38 (13)° (N2—C2—N3) and 126.98 (14)° (N3—C2—C25), which are similar to those in the other reported triazole derivatives (Zhao *et al.*, 2010; Gao *et al.*, 2011). The 1,2,4-triazole ring forms dihedral angles of 114.0 (2), 114.9 (2), 151.9 (2)°  and 149.9 (2)° with the phenyl rings C6—C11, C12—C17 and C25—C30 and the nitrophenyl ring C19—C24. The phenyl ring of the benzyl group attached to atom N1 of the triazole ring (C12—C17) is almost perpendicular to the nitrophenyl ring, with a dihedral angle of 95.1 (2)°.

### Experimental

The title compound was synthesized in the reaction of 4-nitrobenzaldehyde (2.0 mmol) and 3-(4-amino-3-phenyl-5-thioxo-4,5- dihydro-1*H*-1,2,4-triazol-1-yl)-1,3-diphenylpropan-1-one (2.0 mmol), by refluxing in ethanol. The reaction progress was monitored *via* TLC. The resulting precipitate was filtered off, washed with cold ethanol, dried and purified to give the target product as a colorless solid in 66% yield. Crystals suitable for single-crystal X-ray analysis were grown by slow evaporation of a solution in chloroform–ethanol (1:1).

### Refinement

Hydrogen atoms were positioned geometrically and refined as riding (C—H = 0.95-1.00 Å) and allowed to ride on their parent atoms, with *U*_iso_(H) = 1.2*U*_eq_(C).

### Crystal data

**Table:**

C_30_H_23_N_5_O_3_S	*F*(000) = 1112
*M**_r_* = 533.59	*D*_x_ = 1.326 Mg m^−^^3^
Monoclinic, *P*2_1_/*c*	Mo *K*α radiation, λ = 0.71073 Å
Hall symbol: -P 2ybc	Cell parameters from 8592 reflections
*a* = 13.3303 (13) Å	θ = 1.5–27.9°
*b* = 21.832 (2) Å	µ = 0.16 mm^−^^1^
*c* = 9.2773 (9) Å	*T* = 113 K
β = 98.213 (3)°	Prism, colorless
*V* = 2672.2 (4) Å^3^	0.20 × 0.18 × 0.12 mm
*Z* = 4	

### Data collection

**Table:**

Rigaku Saturn CCD area-detector diffractometer	6372 independent reflections
Radiation source: rotating anode	5124 reflections with *I* > 2σ(*I*)
multilayer	*R*_int_ = 0.040
Detector resolution: 14.63 pixels mm^-1^	θ_max_ = 27.9°, θ_min_ = 1.5°
φ and ω scans	*h* = −15→17
Absorption correction: multi-scan (*CrystalClear*; Rigaku/MSC, 2005)	*k* = −27→28
*T*_min_ = 0.968, *T*_max_ = 0.981	*l* = −12→12
27241 measured reflections	

### Refinement

**Table:**

Refinement on *F*^2^	Primary atom site location: structure-invariant direct methods
Least-squares matrix: full	Secondary atom site location: difference Fourier map
*R*[*F*^2^ > 2σ(*F*^2^)] = 0.049	Hydrogen site location: inferred from neighbouring sites
*wR*(*F*^2^) = 0.131	H-atom parameters constrained
*S* = 1.10	*w* = 1/[σ^2^(*F*_o_^2^) + (0.0667*P*)^2^ + 0.1147*P*] where *P* = (*F*_o_^2^ + 2*F*_c_^2^)/3
6372 reflections	(Δ/σ)_max_ < 0.001
352 parameters	Δρ_max_ = 0.33 e Å^−^^3^
0 restraints	Δρ_min_ = −0.25 e Å^−^^3^

### Special details

**Table:**

Geometry. All e.s.d.'s (except the e.s.d. in the dihedral angle between two l.s. planes) are estimated using the full covariance matrix. The cell e.s.d.'s are taken into account individually in the estimation of e.s.d.'s in distances, angles and torsion angles; correlations between e.s.d.'s in cell parameters are only used when they are defined by crystal symmetry. An approximate (isotropic) treatment of cell e.s.d.'s is used for estimating e.s.d.'s involving l.s. planes.
Refinement. Refinement of *F*^2^ against ALL reflections. The weighted *R*-factor *wR* and goodness of fit *S* are based on *F*^2^, conventional *R*-factors *R* are based on *F*, with *F* set to zero for negative *F*^2^. The threshold expression of *F*^2^ > σ(*F*^2^) is used only for calculating *R*-factors(gt) *etc*. and is not relevant to the choice of reflections for refinement. *R*-factors based on *F*^2^ are statistically about twice as large as those based on *F*, and *R*- factors based on ALL data will be even larger.

### Fractional atomic coordinates and isotropic or equivalent isotropic displacement parameters (Å^2^)

**Table:**

	*x*	*y*	*z*	*U*_iso_*/*U*_eq_	
S1	0.40778 (3)	0.258871 (19)	0.46718 (5)	0.03324 (14)	
O1	0.33880 (9)	0.08657 (5)	0.76721 (14)	0.0346 (3)	
O2	−0.05157 (12)	0.41949 (7)	−0.22517 (19)	0.0649 (5)	
O3	−0.07948 (10)	0.33342 (7)	−0.34027 (17)	0.0545 (4)	
N1	0.41855 (9)	0.13669 (6)	0.49993 (14)	0.0250 (3)	
N2	0.37677 (10)	0.08227 (6)	0.44644 (15)	0.0273 (3)	
N3	0.30586 (9)	0.16063 (6)	0.32011 (14)	0.0265 (3)	
N4	0.23159 (10)	0.18795 (7)	0.22006 (15)	0.0305 (3)	
N5	−0.03993 (11)	0.36460 (8)	−0.23724 (19)	0.0440 (4)	
C1	0.37777 (12)	0.18629 (7)	0.42770 (17)	0.0258 (3)	
C2	0.30761 (12)	0.09772 (7)	0.33837 (18)	0.0268 (4)	
C3	0.49357 (11)	0.13916 (7)	0.63312 (17)	0.0244 (3)	
H3	0.4677	0.1679	0.7033	0.029*	
C4	0.50513 (11)	0.07593 (7)	0.70349 (17)	0.0252 (3)	
H4A	0.5231	0.0462	0.6307	0.030*	
H4B	0.5621	0.0772	0.7846	0.030*	
C5	0.41174 (12)	0.05325 (7)	0.76135 (17)	0.0255 (3)	
C6	0.41222 (11)	−0.01130 (7)	0.81389 (16)	0.0246 (3)	
C7	0.50162 (12)	−0.04455 (7)	0.84342 (17)	0.0264 (3)	
H7	0.5640	−0.0265	0.8279	0.032*	
C8	0.50025 (13)	−0.10395 (7)	0.89538 (18)	0.0306 (4)	
H8	0.5617	−0.1265	0.9151	0.037*	
C9	0.40986 (13)	−0.13060 (8)	0.91858 (19)	0.0332 (4)	
H9	0.4091	−0.1715	0.9534	0.040*	
C10	0.32011 (14)	−0.09748 (8)	0.89083 (19)	0.0342 (4)	
H10	0.2580	−0.1155	0.9079	0.041*	
C11	0.32128 (12)	−0.03833 (8)	0.83847 (18)	0.0305 (4)	
H11	0.2598	−0.0159	0.8190	0.037*	
C12	0.59542 (11)	0.16257 (7)	0.60266 (17)	0.0244 (3)	
C13	0.64074 (12)	0.14022 (8)	0.48742 (18)	0.0305 (4)	
H13	0.6057	0.1114	0.4219	0.037*	
C14	0.73711 (13)	0.15985 (8)	0.4674 (2)	0.0363 (4)	
H14	0.7679	0.1442	0.3888	0.044*	
C15	0.78809 (13)	0.20224 (8)	0.5621 (2)	0.0358 (4)	
H15	0.8536	0.2160	0.5482	0.043*	
C16	0.74310 (13)	0.22440 (8)	0.67691 (19)	0.0335 (4)	
H16	0.7783	0.2532	0.7424	0.040*	
C17	0.64733 (12)	0.20516 (7)	0.69740 (18)	0.0291 (4)	
H17	0.6169	0.2210	0.7761	0.035*	
C18	0.23453 (13)	0.24544 (9)	0.1994 (2)	0.0375 (4)	
H18	0.2862	0.2696	0.2532	0.045*	
C19	0.15729 (12)	0.27428 (9)	0.0914 (2)	0.0363 (4)	
C20	0.09104 (13)	0.24006 (8)	−0.0091 (2)	0.0359 (4)	
H20	0.0912	0.1966	−0.0037	0.043*	
C21	0.02564 (13)	0.26954 (9)	−0.1159 (2)	0.0368 (4)	
H21	−0.0185	0.2469	−0.1858	0.044*	
C22	0.02605 (12)	0.33251 (8)	−0.1185 (2)	0.0356 (4)	
C23	0.08666 (13)	0.36786 (9)	−0.0181 (2)	0.0427 (5)	
H23	0.0828	0.4113	−0.0206	0.051*	
C24	0.15371 (13)	0.33767 (9)	0.0872 (2)	0.0422 (5)	
H24	0.1974	0.3608	0.1568	0.051*	
C25	0.24185 (12)	0.05265 (8)	0.25380 (19)	0.0321 (4)	
C26	0.22312 (14)	−0.00168 (8)	0.3235 (2)	0.0435 (5)	
H26	0.2505	−0.0077	0.4227	0.052*	
C27	0.16458 (16)	−0.04698 (9)	0.2484 (3)	0.0564 (6)	
H27	0.1526	−0.0843	0.2959	0.068*	
C28	0.12366 (15)	−0.03807 (10)	0.1051 (3)	0.0564 (6)	
H28	0.0829	−0.0691	0.0544	0.068*	
C29	0.14132 (14)	0.01515 (10)	0.0354 (2)	0.0497 (6)	
H29	0.1124	0.0210	−0.0633	0.060*	
C30	0.20157 (13)	0.06087 (9)	0.1082 (2)	0.0394 (5)	
H30	0.2151	0.0974	0.0589	0.047*	

### Atomic displacement parameters (Å^2^)

**Table:**

	*U*^11^	*U*^22^	*U*^33^	*U*^12^	*U*^13^	*U*^23^
S1	0.0366 (3)	0.0258 (2)	0.0356 (3)	0.00011 (17)	−0.00091 (18)	0.00255 (18)
O1	0.0319 (6)	0.0328 (6)	0.0388 (7)	0.0046 (5)	0.0042 (5)	0.0019 (5)
O2	0.0617 (10)	0.0478 (9)	0.0783 (12)	0.0076 (8)	−0.0139 (8)	0.0202 (8)
O3	0.0449 (8)	0.0643 (10)	0.0487 (9)	0.0034 (7)	−0.0129 (7)	0.0094 (7)
N1	0.0259 (7)	0.0233 (7)	0.0240 (7)	0.0000 (5)	−0.0030 (5)	−0.0019 (5)
N2	0.0260 (7)	0.0266 (7)	0.0273 (7)	−0.0003 (5)	−0.0034 (5)	−0.0049 (6)
N3	0.0228 (7)	0.0326 (8)	0.0226 (7)	0.0029 (5)	−0.0017 (5)	0.0005 (6)
N4	0.0257 (7)	0.0405 (8)	0.0237 (7)	0.0052 (6)	−0.0020 (5)	0.0046 (6)
N5	0.0315 (8)	0.0484 (10)	0.0501 (11)	0.0016 (7)	−0.0006 (7)	0.0160 (8)
C1	0.0236 (8)	0.0310 (9)	0.0221 (8)	0.0022 (6)	0.0015 (6)	0.0023 (6)
C2	0.0232 (8)	0.0315 (9)	0.0251 (8)	0.0021 (6)	0.0010 (6)	−0.0042 (7)
C3	0.0258 (8)	0.0240 (8)	0.0217 (8)	−0.0005 (6)	−0.0020 (6)	−0.0011 (6)
C4	0.0259 (8)	0.0253 (8)	0.0230 (8)	−0.0001 (6)	−0.0017 (6)	0.0005 (6)
C5	0.0275 (8)	0.0259 (8)	0.0213 (8)	0.0014 (6)	−0.0027 (6)	−0.0023 (6)
C6	0.0281 (8)	0.0258 (8)	0.0191 (8)	−0.0018 (6)	0.0009 (6)	−0.0032 (6)
C7	0.0287 (8)	0.0267 (8)	0.0232 (8)	−0.0023 (6)	0.0018 (6)	−0.0020 (6)
C8	0.0348 (9)	0.0290 (8)	0.0274 (9)	0.0017 (7)	0.0026 (7)	−0.0002 (7)
C9	0.0447 (10)	0.0267 (8)	0.0278 (9)	−0.0052 (7)	0.0041 (7)	0.0005 (7)
C10	0.0352 (9)	0.0357 (9)	0.0321 (10)	−0.0100 (7)	0.0055 (7)	−0.0012 (8)
C11	0.0273 (8)	0.0347 (9)	0.0288 (9)	−0.0013 (7)	0.0019 (7)	−0.0030 (7)
C12	0.0269 (8)	0.0221 (8)	0.0225 (8)	0.0005 (6)	−0.0026 (6)	0.0025 (6)
C13	0.0334 (9)	0.0311 (9)	0.0255 (9)	−0.0055 (7)	−0.0004 (7)	−0.0050 (7)
C14	0.0378 (10)	0.0422 (10)	0.0298 (9)	−0.0041 (8)	0.0085 (7)	−0.0060 (8)
C15	0.0314 (9)	0.0401 (10)	0.0359 (10)	−0.0073 (8)	0.0049 (7)	0.0011 (8)
C16	0.0332 (9)	0.0298 (9)	0.0360 (10)	−0.0072 (7)	−0.0003 (7)	−0.0045 (7)
C17	0.0316 (9)	0.0283 (8)	0.0268 (8)	−0.0016 (7)	0.0015 (7)	−0.0048 (7)
C18	0.0290 (9)	0.0465 (11)	0.0358 (10)	0.0001 (8)	0.0003 (7)	0.0075 (8)
C19	0.0250 (9)	0.0456 (11)	0.0376 (10)	0.0017 (7)	0.0023 (7)	0.0116 (8)
C20	0.0309 (9)	0.0395 (10)	0.0368 (10)	0.0007 (7)	0.0029 (7)	0.0073 (8)
C21	0.0254 (9)	0.0463 (11)	0.0384 (10)	0.0004 (8)	0.0034 (7)	0.0045 (8)
C22	0.0217 (8)	0.0459 (11)	0.0384 (10)	0.0008 (7)	0.0014 (7)	0.0124 (8)
C23	0.0324 (10)	0.0420 (11)	0.0523 (12)	−0.0025 (8)	0.0010 (8)	0.0139 (9)
C24	0.0327 (10)	0.0443 (11)	0.0464 (12)	−0.0056 (8)	−0.0054 (8)	0.0087 (9)
C25	0.0236 (8)	0.0375 (10)	0.0331 (9)	0.0050 (7)	−0.0031 (7)	−0.0122 (8)
C26	0.0400 (11)	0.0356 (10)	0.0498 (12)	0.0003 (8)	−0.0109 (9)	−0.0092 (9)
C27	0.0500 (13)	0.0375 (11)	0.0750 (16)	−0.0032 (9)	−0.0141 (11)	−0.0136 (11)
C28	0.0390 (11)	0.0530 (13)	0.0705 (16)	0.0023 (10)	−0.0148 (10)	−0.0310 (12)
C29	0.0320 (10)	0.0686 (15)	0.0441 (12)	0.0096 (10)	−0.0092 (8)	−0.0259 (11)
C30	0.0292 (9)	0.0521 (11)	0.0348 (10)	0.0064 (8)	−0.0034 (7)	−0.0142 (9)

### Geometric parameters (Å, °)

**Table:**

S1—C1	1.6622 (16)	C12—C17	1.394 (2)
O1—C5	1.2218 (18)	C13—C14	1.392 (2)
O2—N5	1.215 (2)	C13—H13	0.9500
O3—N5	1.229 (2)	C14—C15	1.386 (2)
N1—C1	1.3466 (19)	C14—H14	0.9500
N1—N2	1.3745 (17)	C15—C16	1.382 (2)
N1—C3	1.4754 (18)	C15—H15	0.9500
N2—C2	1.3058 (19)	C16—C17	1.383 (2)
N3—C2	1.383 (2)	C16—H16	0.9500
N3—N4	1.3911 (17)	C17—H17	0.9500
N3—C1	1.3984 (19)	C18—C19	1.471 (2)
N4—C18	1.271 (2)	C18—H18	0.9500
N5—C22	1.483 (2)	C19—C24	1.385 (3)
C2—C25	1.468 (2)	C19—C20	1.405 (2)
C3—C12	1.515 (2)	C20—C21	1.382 (2)
C3—C4	1.525 (2)	C20—H20	0.9500
C3—H3	1.0000	C21—C22	1.375 (2)
C4—C5	1.508 (2)	C21—H21	0.9500
C4—H4A	0.9900	C22—C23	1.379 (3)
C4—H4B	0.9900	C23—C24	1.392 (2)
C5—C6	1.491 (2)	C23—H23	0.9500
C6—C7	1.389 (2)	C24—H24	0.9500
C6—C11	1.396 (2)	C25—C26	1.390 (3)
C7—C8	1.385 (2)	C25—C30	1.392 (2)
C7—H7	0.9500	C26—C27	1.384 (3)
C8—C9	1.382 (2)	C26—H26	0.9500
C8—H8	0.9500	C27—C28	1.377 (3)
C9—C10	1.390 (2)	C27—H27	0.9500
C9—H9	0.9500	C28—C29	1.367 (3)
C10—C11	1.381 (2)	C28—H28	0.9500
C10—H10	0.9500	C29—C30	1.394 (3)
C11—H11	0.9500	C29—H29	0.9500
C12—C13	1.389 (2)	C30—H30	0.9500
			
C1—N1—N2	113.68 (12)	C12—C13—H13	119.8
C1—N1—C3	124.37 (13)	C14—C13—H13	119.8
N2—N1—C3	121.68 (12)	C15—C14—C13	119.95 (16)
C2—N2—N1	105.02 (12)	C15—C14—H14	120.0
C2—N3—N4	120.35 (12)	C13—C14—H14	120.0
C2—N3—C1	108.21 (12)	C16—C15—C14	119.68 (16)
N4—N3—C1	130.87 (13)	C16—C15—H15	120.2
C18—N4—N3	119.33 (14)	C14—C15—H15	120.2
O2—N5—O3	124.84 (16)	C15—C16—C17	120.68 (15)
O2—N5—C22	117.86 (17)	C15—C16—H16	119.7
O3—N5—C22	117.30 (15)	C17—C16—H16	119.7
N1—C1—N3	102.69 (13)	C16—C17—C12	120.07 (16)
N1—C1—S1	126.13 (12)	C16—C17—H17	120.0
N3—C1—S1	131.16 (12)	C12—C17—H17	120.0
N2—C2—N3	110.38 (13)	N4—C18—C19	119.45 (16)
N2—C2—C25	122.63 (15)	N4—C18—H18	120.3
N3—C2—C25	126.98 (14)	C19—C18—H18	120.3
N1—C3—C12	111.99 (12)	C24—C19—C20	119.79 (16)
N1—C3—C4	109.89 (12)	C24—C19—C18	117.69 (16)
C12—C3—C4	110.29 (12)	C20—C19—C18	122.47 (17)
N1—C3—H3	108.2	C21—C20—C19	120.06 (17)
C12—C3—H3	108.2	C21—C20—H20	120.0
C4—C3—H3	108.2	C19—C20—H20	120.0
C5—C4—C3	114.30 (13)	C22—C21—C20	118.34 (17)
C5—C4—H4A	108.7	C22—C21—H21	120.8
C3—C4—H4A	108.7	C20—C21—H21	120.8
C5—C4—H4B	108.7	C21—C22—C23	123.43 (16)
C3—C4—H4B	108.7	C21—C22—N5	118.82 (16)
H4A—C4—H4B	107.6	C23—C22—N5	117.74 (16)
O1—C5—C6	120.97 (15)	C22—C23—C24	117.70 (18)
O1—C5—C4	121.53 (14)	C22—C23—H23	121.2
C6—C5—C4	117.49 (13)	C24—C23—H23	121.2
C7—C6—C11	119.18 (15)	C19—C24—C23	120.59 (17)
C7—C6—C5	121.46 (14)	C19—C24—H24	119.7
C11—C6—C5	119.33 (14)	C23—C24—H24	119.7
C8—C7—C6	120.30 (15)	C26—C25—C30	119.43 (16)
C8—C7—H7	119.8	C26—C25—C2	117.15 (16)
C6—C7—H7	119.8	C30—C25—C2	123.37 (17)
C9—C8—C7	120.23 (16)	C27—C26—C25	120.1 (2)
C9—C8—H8	119.9	C27—C26—H26	120.0
C7—C8—H8	119.9	C25—C26—H26	120.0
C8—C9—C10	119.91 (16)	C28—C27—C26	120.1 (2)
C8—C9—H9	120.0	C28—C27—H27	119.9
C10—C9—H9	120.0	C26—C27—H27	119.9
C11—C10—C9	119.93 (16)	C29—C28—C27	120.39 (19)
C11—C10—H10	120.0	C29—C28—H28	119.8
C9—C10—H10	120.0	C27—C28—H28	119.8
C10—C11—C6	120.44 (16)	C28—C29—C30	120.36 (19)
C10—C11—H11	119.8	C28—C29—H29	119.8
C6—C11—H11	119.8	C30—C29—H29	119.8
C13—C12—C17	119.23 (15)	C25—C30—C29	119.60 (19)
C13—C12—C3	121.66 (13)	C25—C30—H30	120.2
C17—C12—C3	119.01 (14)	C29—C30—H30	120.2
C12—C13—C14	120.38 (15)		
			
C1—N1—N2—C2	−0.32 (18)	N1—C3—C12—C17	136.38 (14)
C3—N1—N2—C2	173.93 (14)	C4—C3—C12—C17	−100.89 (16)
C2—N3—N4—C18	−176.27 (16)	C17—C12—C13—C14	0.4 (2)
C1—N3—N4—C18	13.5 (3)	C3—C12—C13—C14	−176.07 (15)
N2—N1—C1—N3	−0.65 (17)	C12—C13—C14—C15	−0.4 (3)
C3—N1—C1—N3	−174.72 (13)	C13—C14—C15—C16	0.5 (3)
N2—N1—C1—S1	177.81 (12)	C14—C15—C16—C17	−0.6 (3)
C3—N1—C1—S1	3.7 (2)	C15—C16—C17—C12	0.6 (2)
C2—N3—C1—N1	1.33 (17)	C13—C12—C17—C16	−0.5 (2)
N4—N3—C1—N1	172.49 (15)	C3—C12—C17—C16	176.08 (14)
C2—N3—C1—S1	−177.02 (14)	N3—N4—C18—C19	178.74 (15)
N4—N3—C1—S1	−5.9 (3)	N4—C18—C19—C24	170.56 (18)
N1—N2—C2—N3	1.19 (18)	N4—C18—C19—C20	−11.9 (3)
N1—N2—C2—C25	−177.76 (15)	C24—C19—C20—C21	2.9 (3)
N4—N3—C2—N2	−173.91 (13)	C18—C19—C20—C21	−174.62 (17)
C1—N3—C2—N2	−1.65 (19)	C19—C20—C21—C22	−1.3 (3)
N4—N3—C2—C25	5.0 (2)	C20—C21—C22—C23	−1.6 (3)
C1—N3—C2—C25	177.24 (16)	C20—C21—C22—N5	176.85 (16)
C1—N1—C3—C12	−69.61 (19)	O2—N5—C22—C21	168.08 (18)
N2—N1—C3—C12	116.77 (15)	O3—N5—C22—C21	−11.4 (3)
C1—N1—C3—C4	167.44 (14)	O2—N5—C22—C23	−13.4 (3)
N2—N1—C3—C4	−6.2 (2)	O3—N5—C22—C23	167.13 (17)
N1—C3—C4—C5	−65.96 (17)	C21—C22—C23—C24	2.8 (3)
C12—C3—C4—C5	170.10 (12)	N5—C22—C23—C24	−175.60 (17)
C3—C4—C5—O1	−8.9 (2)	C20—C19—C24—C23	−1.6 (3)
C3—C4—C5—C6	172.12 (13)	C18—C19—C24—C23	176.04 (18)
O1—C5—C6—C7	−163.09 (15)	C22—C23—C24—C19	−1.2 (3)
C4—C5—C6—C7	15.9 (2)	N2—C2—C25—C26	26.7 (2)
O1—C5—C6—C11	14.8 (2)	N3—C2—C25—C26	−152.10 (18)
C4—C5—C6—C11	−166.23 (14)	N2—C2—C25—C30	−150.80 (17)
C11—C6—C7—C8	0.6 (2)	N3—C2—C25—C30	30.4 (3)
C5—C6—C7—C8	178.48 (15)	C30—C25—C26—C27	−0.2 (3)
C6—C7—C8—C9	−0.1 (2)	C2—C25—C26—C27	−177.80 (17)
C7—C8—C9—C10	−0.5 (3)	C25—C26—C27—C28	−0.8 (3)
C8—C9—C10—C11	0.8 (3)	C26—C27—C28—C29	0.7 (3)
C9—C10—C11—C6	−0.4 (3)	C27—C28—C29—C30	0.4 (3)
C7—C6—C11—C10	−0.3 (2)	C26—C25—C30—C29	1.4 (3)
C5—C6—C11—C10	−178.26 (15)	C2—C25—C30—C29	178.79 (16)
N1—C3—C12—C13	−47.16 (19)	C28—C29—C30—C25	−1.5 (3)
C4—C3—C12—C13	75.57 (18)		
